# In silico analysis of overall survival with *YBX1* in male and female solid tumours

**DOI:** 10.1038/s41598-024-57771-y

**Published:** 2024-03-27

**Authors:** David Robert Grimes, Treewut Rassamegevanon, Laure Marignol

**Affiliations:** 1 Applied Radiation Therapy Trinity, Discipline of Radiation Therapy, Trinity College Dublin, Dublin, Ireland; 2 OncoRay-National Center for Radiation Research in Oncology, Faculty of Medicine and University Hospital Carl Gustav Carus, Technische Universität Dresden, Helmholtz-Zentrum Dresden–Rossendorf, Dresden, Germany; 3 German Cancer Consortium (DKTK), Partner Site Dresden, Dresden, Germany; 4 German Cancer Research Center (DKFZ), Heidelberg, Germany

**Keywords:** Y-box-binding protein 1, Survival, Cancer, Biological sex, Cancer, Computational biology and bioinformatics, Genetics

## Abstract

The Y-box binding protein-1 (*YBX1*) gene codes for a multifunctional oncoprotein that is increasingly being linked to the regulations of many aspects of cancer cell biology. Disparities in treatment outcomes between male and female cancer patients are increasingly reported. This study aimed to examine the relationship between *YBX1* expression and overall survival in male and female patients with solid tumours. Overall survival and *YBX1* expression data for cohorts of male and female cancer patients obtained from freely available databases were analysed with a cox proportional hazard model with covariates of biological sex and *YBX1* expression. Kaplan–Meier curves and Violin plots were constructed for segregated male and female cohorts. High *YBX1* expression was significantly associated with poor survival in 2 female-only and 4 mixed-sex cancer sites. In female lung cancer patients, better survival and lower YBX*1* expression were identified. The clinical importance of *YBX1* expression in cancer ought to be evaluated in a sex-specific manner, especially in lung cancer.

## Introduction

YB-1, also known as DNA binding protein B (DBPB), is one of three members of the Y-box family of transcription factors^1^ whose impact on cancer cell biology is increasingly supported by experimental studies identifying promotion of cell proliferation and apoptosis^2^, regulation of DNA proliferation and repair^3^, stemness^4^ and response to treatment^5^. Meanwhile, the consideration of sex as a biological variable in cancer research is identifying differences in cancer cell biology mechanisms between the sexes^6^. YB-1 was reported to interact with the X-linked ribosomal protein S4 (RPS4X), driving cisplatin sensitivity in breast cancer cell lines^7^, and poor outcomes in ovarian^8^ and bladder cancer^9^. But how biological sex relate to the biological and clinical impact of major regulators of cancer cell biology such as YB-1 remains unknown.

The human YB-1 gene (*YBX1*) is located on chromosome 1 (1p34), contains eight exons and spans 19 kb of genomic DNA. The YB-1 gene promoter contains several E-boxes and CG-repeats that are important for YB-1 transcription into a 1.5 kb-long mRNA^10^ and codes for a 324 amino acid YB-1 protein normally localised in the cytoplasm where it plays a key role in the regulation of mRNA translation^11,12^. The detection of this protein is rapidly emerging as both a clinically useful diagnostic biomarker and a potentially viable therapeutic target in many cancer types^13,14^. The analysis of *YBX1* mRNA levels in Head and Neck cancer patients linked high expression with poor prognosis^15^. But the clinical importance of the mRNA expression levels of this oncoprotein remains poorly investigated.

The Sex as a Biological Variable (SABV) policy established by the US National Institute of Health^16^ requires researchers to distinguish between “sex”, a term related to the presence of XX or XY chromosomes in humans, from “gender”, a term associated with the social, cultural and psychological traits of human males and females. Analysis of the Cancer Genome Atlas identified sex-biased signatures in 53% of clinically actionable genes (60/114) investigated^17^. Differences between the sexes are increasingly documented in the functions of both the innate and adaptive immune systems^18^, regulation of miRNAs and mRNA^17,19^, genetic polymorphism in antibody responses^20^, and the microbiome^21^. Mice studies have reported sex-specific cell death programs with males prone to PARP-1 necrosis and females to caspase-dependent apoptosis^22^. Others identified differences in basal redox state^23^, response to oxidative stress^24^, sensitivity to both apoptosis and autophagy^25^.

Taking *YBX1* mRNA levels as a test case, this in silico study aimed to examine whether the segreration of cohorts of patients with solid cancer that commonly develop in both males and females according to their recorded biological sex could identify novel associations between expression and overall survival.

## Materials and methods

### Patient cohorts

*YBX1* mRNA expression profiles and survival data of patients diagnosed with 13 cancer types that commonly develop in both males and females were accessed from various databases (Table 1). The *YBX1* mRNA expression profiles and survival data of female patients with breast, ovarian and uterine endometrial cancer were included as examples of disease site where sex is a controllable biological variable. Cancer cohorts were chosen to have a minimum of 150 subjects per condition, and recorded events (deaths) ranging from 15.8% (Rectum adenocarcinoma) to 61.7% of the sample (Ovarian cancer) allowing for robust survival analysis of overall survival (OS). This data was downloaded via the KM-Plotter web-interface (http://www.kmplot.com)^26^, and imported in the RStudio software for analysis.

**Table 1:** Cancer cohorts and sources.

Cancer cohort	Database/s	Sample size (sex division)	mRNA Expression quantification technique
Bladder cancer	Pancancer^27^ (derived from TCGA repository)	N = 406 (m = 298, f = 108)	mRNA sequence
Breast cancer	GEO repository^28^	N = 4929 (m = 0, f = 4929)	Genechip
Cervical squamous cell carcinoma	Pancancer^27^ (derived from TCGA repository)	N = 304 (m = 0, f = 304)	mRNA sequence
Gastric cancer	Gastric cancer database^29^	N = 780 (m = 544, f = 236)	Genechip
Head-neck squamous cell carcinoma	Pancancer^27^ (derived from TCGA repository)	N = 499 (m = 366, f = 133)	mRNA sequence
Liver hepatocellular carcinoma	Pancancer^27^ (derived from TCGA repository)	N = 370 (m = 249, f = 141)	mRNA sequence
Lung cancer	caBIG/GEO/TCGA repositories^30^	N = 1814 (m = 1100, f = 714)	Genechip
Ovarian cancer	GEO/Cancer Atlas^31^	N = 1435 (m = 0, f = 1435)	Genechip
Pancreatic ductal adenocarcinoma	Pancancer^27^ (derived from TCGA repository)	N = 177 (m = 97, f = 80)	mRNA sequence
Renal clear cell carcinoma	Pancancer^27^ (derived from TCGA repository)	N = 530 (m = 344, f = 186)	mRNA sequence
Renal papillary cell carcinoma	Pancancer^27^ (derived from TCGA repository)	N = 287 (m = 211, f = 76)	mRNA sequence
Rectum adenocarcinoma	Pancancer^27^ (derived from TCGA repository)	N = 165 (m = 90, f = 75)	mRNA sequence
Sarcoma	Pancancer^27^ (derived from TCGA repository)	N = 259 (m = 118, f = 141)	mRNA sequence
Stomach adenocarcinoma	Pancancer^27^ (derived from TCGA repository)	N = 371 (m = 238, f = 133)	mRNA sequence
Uterine corpus endometrial carcinoma	Pancancer^27^ (derived from TCGA repository)	N = 542 (m = 0, f = 542)	mRNA sequence

### Analysis of overall survival with *YBX1* and biological sex

The impact of both YB-1 and sex on cancer survival for the 15 cancer types was tested with a cox proportional hazard model comprising of three co-variates: YB-1 mRNA levels (a continuous measure), sex (a categorical variable), and their interaction term. This analysis was implemented in RStudio (2022.02.2 + 485 "Prairie Trillium" Release) employing the *survival* package, with the relevant code provided in the supplementary material (S1). In convention with best statistical practice^32^, all covariates were tested simultaneously. Subgroup analysis was explicitly avoided unless significant interaction between covariates was detected. A Benjamini–Hochberg procedure^33^ was employed to correct for multiple testing in the 15 cancer types tested and ensure a Family-wise error rate of $\alpha =0.05$.

### Analysis of *YBX1* expression according to biological sex

*YBX1* expression distribution between male and female sexes was analysed with a two-sample t-test. The Šidák variation of the Bonferroni correction^34^ was implemented to correct for multiple comparison. The threshold significance was set by solving ${a}_{s}=1-\sqrt[m]{1-\alpha }$, where *m* is the number of cancer types analysed and $\alpha =0.05.$

### Kaplan Meier survival analysis

Kaplan Meier plots were constructed for cancers with significant differences after the Benjamini–Hochberg procedure was employed, to investigate sex related differences, and a significance test performed using the survival package in R implemented through RStudio (2022.02.2 + 485 "Prairie Trillium" Release, R-Version 4.2.3, https://www.r-project.org/) employing the survival package, with the relevant code provided in the supplementary material S1.

### Co-expression analysis

Co-expression coefficients were generated by downloading data from the “Coexpression tab” in the cBioportal web service. The list of genes located on chromosome X was downloaded from Uniprot and validated in Human Genome Organisation (HUGO) database. The chromosome X genes that showed a Spearman’S correlation coefficient greater or lower than 0.25 and − 0.25, respectively, and q value < 0.05 (FDR < 0.5) were deemed correlated with YB-1.

### Ethics declaration

All data generated or analysed during this study was downloaded from the freely accessible databases outlined in Table 1. This data was irrevocably anonymous and is deposited on these open access platforms. All methods were carried out in accordance with relevant guidelines and regulations.

## Results

### High *YBX1* expression is associated with reduced overall survival

**Figure 1:**
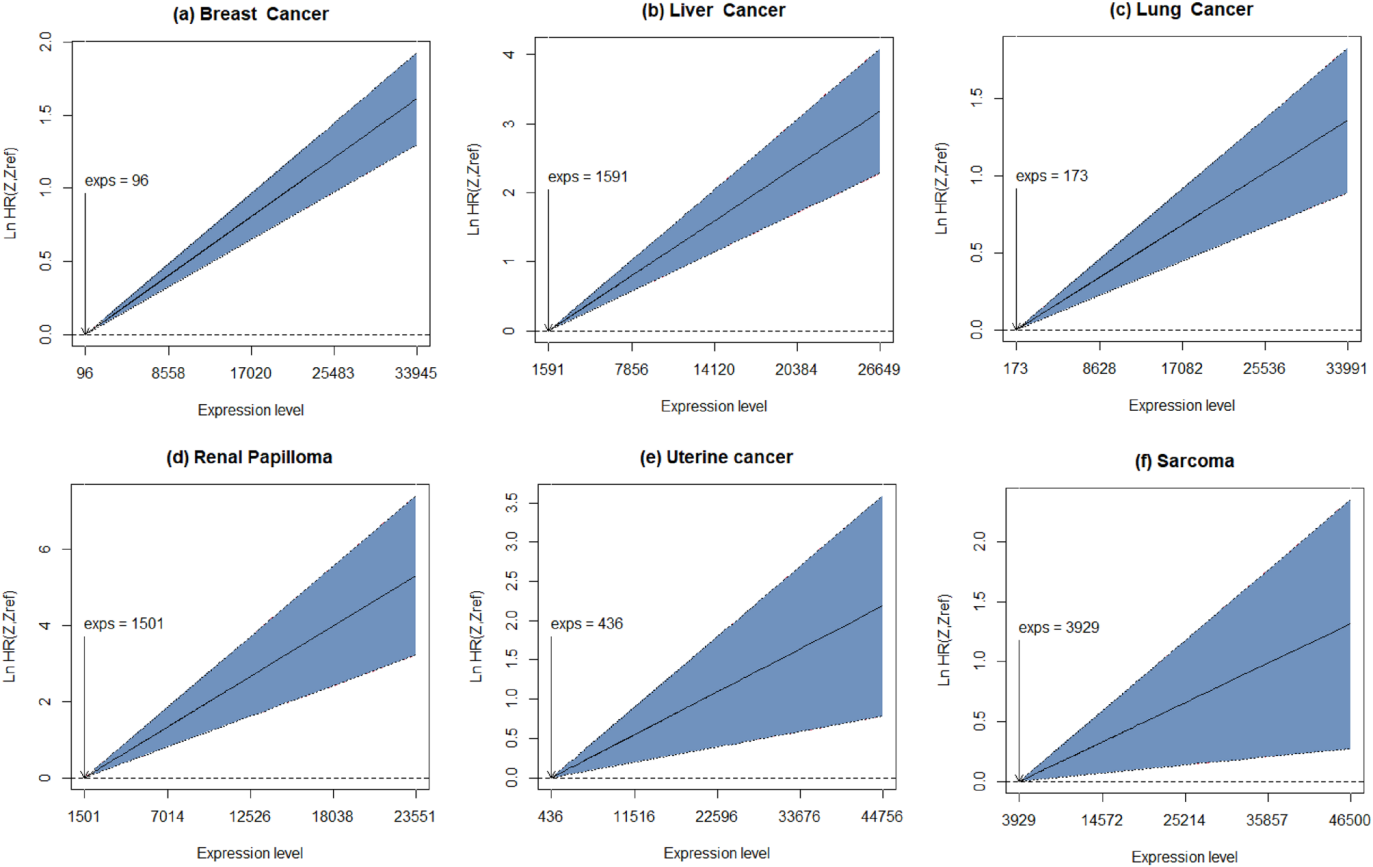
Log of the Hazard ratio against YB-1 expression levels for cancers with significant expression effects in Table 2. The shaded region depicts the 95% confidence interval. Note the varying axes limits for both log hazard ratio and expression level.

We first analysed available survival data in all 15 identified cancer cohorts using a Cox proportional hazard model. Application of the Benjamini–Hochberg procedure to keep the false discovery rate at $\alpha =0.05$ for all cohorts yielded a threshold significance value of ${\alpha }_{bh}=0.02$. A significant relationship between *YBX1* expression and survival was detected in 6 cancer types: breast, liver, lung, renal papilloma, uterine cancer, and sarcoma (Table 2). In all these sites, the hazard ratio for *YBX1* was > 1, indicating that higher expression levels were associated with poorer survival (Table 2 and Fig. 1). As expected, biological sex did not affect survival in female only cancers (Breast, uterine cancer). In cancer sites affecting both males and females, biological sex did not interact with *YBX1* expression and did not affect survival in this analysis (Hazard ratio = 1) (data not shown).

**Table 2:** YBX1 expression and biological sex survival analysis in 15 cancer cohorts.

Cancer cohort	Sample size (sex division)	YBX1 expression profile (mean, min–max values)	YBX1 expression significance level	YBX1 hazard ratio* (95% confidence)
Significant after Benjamini–Hochberg procedure for multiple comparisons				
Breast cancer	N = 4929 (m = 0, f = 4929)	GeneChip (9370, 96–33945)	$p <2 \times {10}^{-16}$	1.049 (1.039–1.059)
Liver cancer	N = 370 (m = 249, f = 141)	mRNA sequence (6649, 1591–26649)	$p=1.45 \times {10}^{-11}$	1.145 (1.110–1.191)
Lung cancer	N = 1814 (m = 1100, f = 714)	GeneChip (10975, 173–33991)	$p=2.41 \times {10}^{-6}$	1.038 (1.022–1.054)
Renal papilloma	N = 287 (m = 211, f = 76)	mRNA sequence (7743, 1501–23551)	$p=9.50 \times {10}^{-6}$	1.287 (1.151–1.439)
Uterine cancer	N = 542 (m = 0, f = 542)	mRNA sequence (13243, 436–44756)	$p=0.00228$	1.051 (1.018–1.084)
Sarcoma	N = 259 (m = 118, f = 141)	mRNA sequence (14586, 3929–46500)	$p=0.00359$	1.089 (1.028–1.154)
Non-significant after Benjamini**–**Hochberg procedure for multiple comparisons				
Stomach cancer	N = 371 (m = 238, f = 133)	mRNA sequence (12902, 3259–29802)	$p=0.0262$	0.994 (0.898–0.993)
Ovarian cancer	N = 1435 (m = 0, f = 1435)	GeneChip (15042, 170–43850)	$p=0.0626$	1.009 (0.999–1.020)
Cervical cancer	N = 304 (m = 0, f = 304)	mRNA sequence (13757, 2707–40652)	$p=0.115$	1.041 (0.990–1.094)
Bladder cancer	N = 406 (m = 298, f = 108)	mRNA sequence (13457, 1223–192954)	$p=0.134$	1.007 (0.998–1.016)
Renal clear cell	N = 530 (m = 344, f = 186)	mRNA sequence (7975, 861–19403)	$p=0.156$	1.063 (0.977–1.157)
Rectal cancer	N = 165 (m = 90, f = 75)	mRNA sequence (14727, 4704–31516)	$p =0.203$	0.923 (0.816–1.044)
Pancreatic cancer	N = 177 (m = 97, f = 80)	mRNA sequence (8027, 2463–38335)	$p= 0.475$	1.019 (0.967–1.074)
Gastric cancer	N = 780 (m = 544, f = 236)	GeneChip (12443, 3167–25195)	$p=0.882$	1.003 (0.968–1.039)
Head and neck cancer	N = 499 (m = 366, f = 133)	mRNA sequence (13105, 3534–56844)	$p= 0.976$	1.000 (0.971–1.031)

*Hazard ratios are given per 1000 units of gene expression Quoted p-values and hazard ratios refer to YB-1 expression. Direct sex effects did not reach significance threshold and are not included here.

### *YBX1* expression and biological sex

**Figure 2:**
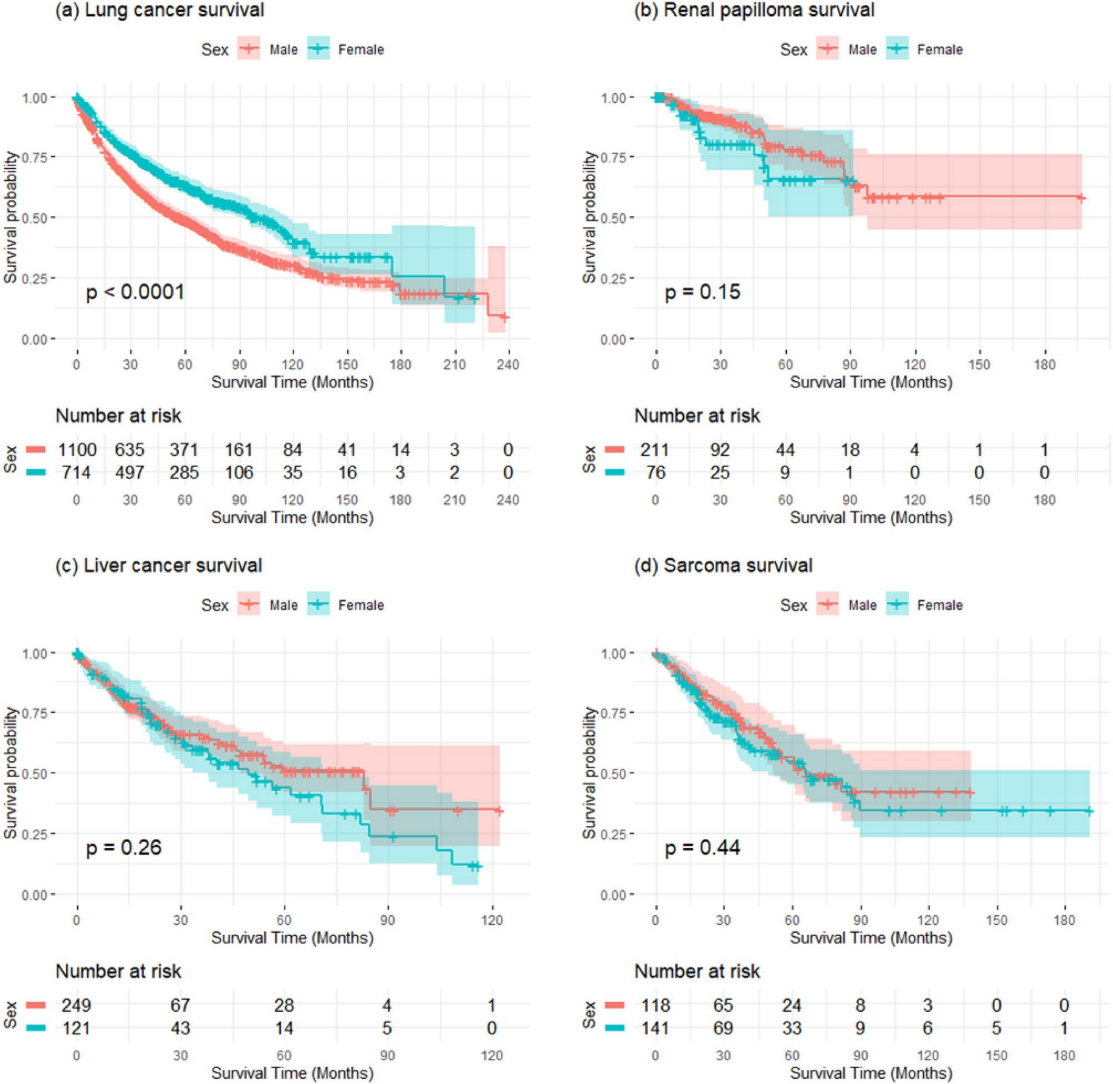
Kaplan–Meier survival curves for male and female patients cohorts in lung, renal papilloma, liver and sarcoma. The p-values for the differences between male and female cohort survival is given in the figure for each cancer type.

We next focused on the 4 cancer sites that affect both males and females, where *YBX1* expression was identified to significantly affect survival: liver, lung, renal papilloma, and sarcoma. First, we constructed Kaplan–Meier curves to compare the survival of segregated male and female patient cohorts (Fig. 2). Lung was the only cancer type displaying a highly significant difference in survival when the data was analysed according to sex.

**Figure 3:**
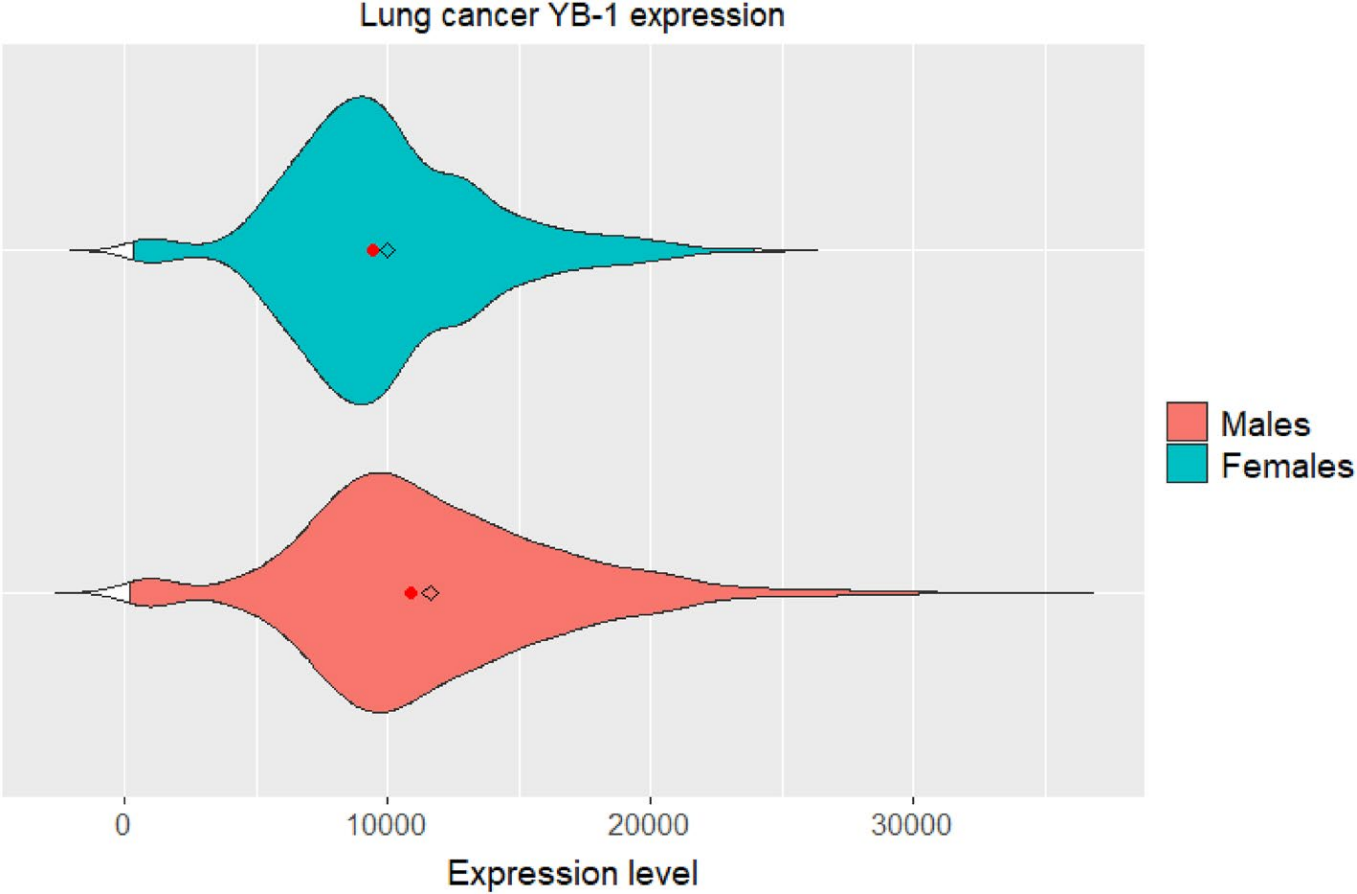
Sex differences in YBX1 expression distribution for Lung cancer. The red dot indicates distribution medians, and the diamond indicates distribution means.

Second, we examined the distributions of *YBX1* expression levels in both male and female cohorts and confirmed that these were approximately normal, with two-tailed t-tests. Finally, we compared *YBX1* expression between sexes (Table 3). A Šidák variation of the Bonferroni correction yielded a threshold of ${\alpha }_{bh}=0.0127$. At this threshold, sex differences in *YBX1* expression for the Lung cancer cohort were highly significant with a Cohen’s D of 0.363, indicating a medium to large effect size. A violin plot of the *YBX1* expression distribution indicates higher expression in males, compared to females in lung cancer (Fig. 3).

**Table 3:** Sex differences in YB-1 expression.

Cancer type	Sex difference in expression significance	Cohen’s D
Significant after Šidák correction for multiple comparisons		
Lung cancer	$p=2.545 \times {10}^{-14}$	0.363
Non-significant after Šidák correction for multiple comparisons		
Renal papilloma	$p=0.0667$	0.245
Liver cancer	$p=0.0158$	0.267
Sarcoma	$p=0.193$	0.162

### *YBX1* expression and the X chromosome

**Figure 4:**
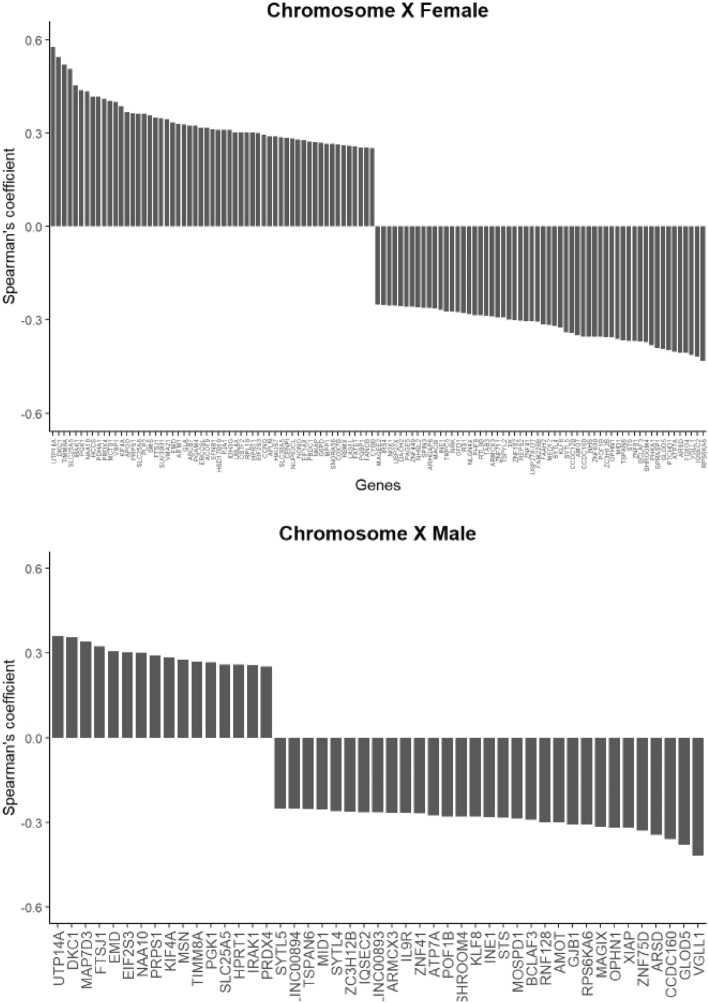
Waterfall plots of the X-linked genes identified as associated with YB-1 on male and female bladder cancer patients.

We next focused on one cancer site that is known to affect both males and females differently^35–38^, where *YBX1* expression was not identified to significantly affect survival in our analysis: bladder cancer. We generated correlation coefficients for the expression of *YBX1* and individual X-linked genes in both male and female patients. In total 47 (male) and 115 (female) chromosome X genes were identified to co-express positively or negatively with *YBX1* (Fig. 4). N = 37 were common to both sexes (Supplementary material S2). Of those, DKC1 held the highest positive correlation coefficient (0.36) and VGLL1 the lowest (− 0.42). Kaplan–Meier analysis identified an association between expression and overall survival in both male and female cohort for VGLL1 but not DKC1 (data not shown). Of the 78 genes uniquely identified in the female cohort (Supplementary material S2), VBP1 held the highest positive correlation coefficient (0.4) and FOXO4 the lowest (− 0.40). In these female patients, low VBP1 was associated with poorer overall survival (HR = 1.87 (1.05–3.01), p = 0.03). No association was detected for FOXO4. In the male cohort (Supplementary material S2), all but MOSPD1 appeared associated with overall survival on Kaplan–Meier analysis (data not shown).

## Discussion

Sex is a fundamental biological variable increasingly studied as a factor influencing cancer treatment response^39^. Cancer affects men and women^40^; but we treat patients. This sex-neutral approach results from the belief that circulating sex hormones dominate sexual differentiation biology^41^ and the practice of sex data pooling^39^. Our efforts, however, yield unequal success between the sexes^42^. The predicted rise in the 19.3 million annual new cancer cases^40^ will worsen the clinical and societal impact of treatment resistance and innovation in cancer management is a clinical priority.

YB-1 is a multifunctional protein involved in both the transcriptional and translational regulation of gene expression^43^. The detection of this oncoprotein in tumour specimens is increasingly linked to poor patient outcomes. But the importance of *YBX1* gene expression remains poorly documented. In the Prognoscan database^44^, YBX1 expression is associated with an increased hazard ratio for overall survival with Breast cancer, Lung cancer and prostate cancer (data not shown). We used available data for 15 cancer types to examine the link between *YBX1* expression and survival outcomes. Our analysis identifies that high *YBX1* expression is associated with poor survival in 6 cancer types.

YB-1 controls almost all DNA and mRNA dependent processes in the cell such as cellular differentiation, proliferation and stress response^43^. The regulation of these critical processes is increasingly linked to biological sex. This fundamental biological variable is defined by the presence of genetic information provided by the X and Y chromosomes, whose regulation and loss are proving relevant to cancer biology and treatment outcomes^7–9,36,38,45–47^. Lack of sex analysis in preclinical and interventional studies was proposed to increase the risk for an effect being lost or claimed where it only applies to one sex^16^. In biomedical research analysis of the literature identified the underrepresentation of female animals and a lack of sex-specific reporting^48^. Our analysis expands earlier report that a correlation between the expression of *YB-1* and X-linked genes exists^7–9^. In both male and female patients with bladder cancer, we identified 37 interactions common to both sexes, 10 limited to male patients and 78 to female patients. The relevance of biological sex in this disease is increasingly reported and could affect disease classification, and immune responses^36,38,47^. Further characterisation of the relevance of X-linked genes to the behaviour of malignant diseases is warranted.

This study aimed to determine whether the relationship between *YBX1* expression and overall survival is affected by the biological sex categorisation of the patient cohorts^49^. Cox proportional hazard analysis of available data failed to identify biological sex as a co-variate significantly affecting survival in all 15 cancer sites examined. Similarly, meta-analysis of YB-1 protein expression, survival and clinicopathological features indicated that overexpression correlates with worse overall survival, but no association was identified with sex on multi-variate analysis^13^. Yet, Kaplan Meier curves were significantly different between male and female lung cancer patients. In lung cancer, the analysis of gene expression signatures according to the sex of the patients included revealed distinct cluster groups^50^. Our analysis of *YBX1* expression identified a significant difference between the expression distributions of the male and the female cohorts in the case of lung cancer, which might be related to the stark differences in mortality between sexes. While we were unable to find a suitable data set for male-specific disease like prostate cancer, the prognoscan database^44^ suggests that YBX1 expression increases hazard ratio in prostate cancer survival, and future work is needed to elucidate why this might be the case.

This work serves to highlight that the generation of sex‐based analysis could refine the relevance of candidate genetic markers and emerging therapeutic targets. Further evaluation of the biological and clinical implications of our findings is needed. Future studies aimed as assessing he biological functions and clinical importance of *YBX1*, and its protein product in cancer ought to consider the biological sex of their models and patients, especially in lung cancer. This could be of particular relevance to the development of senolytic drugs, such as the YB-1 inhibitor fisetin, for the treatment of cancer^51^. In lung cancer, several reports already indicate the capacity of this drug to affect lung cancer cell growth, migration and apoptosis^52–54^, but unfortunately this effect was only tested in male lung cancer models.

### Supplementary Information

Supplementary Information.

Supplementary Table 1.

Supplementary Table 2.

Supplementary Table 3.

## Data Availability

The datasets used and/or analysed during the current study available from the corresponding author on reasonable request.
