# Supplementary material for: In silico analysis of overall survival with YBX1 in male and female solid tumours
Source: Sci Rep. 2024 Mar 27;14:7218. doi: 10.1038/s41598-024-57771-y (PMC10973514; doi:10.1038/s41598-024-57771-y)
Supplement: Supplementary file 1 — Supplementary Information. [file 41598_2024_57771_MOESM1_ESM.docx]

Supplementary Material 1: Code used to test the impact of both YB-1 and sex on cancer survival for the 15 cancer types with a cox proportional hazard model comprising of three co-variates: YB-1 mRNA levels (a continuous measure), sex (a categorical variable), and their interaction term. in RStudio (2022.02.2+485 "Prairie Trillium" Release)

library(ggplot2)

library(smoothHR)

library(rms)

library(visreg)

library(survival)

library(survminer)

library(dplyr)

library(ggfortify)

rm(list = ls())

#import data manually - open excel file of gender, cancer, and expression data with time to outcomes

#this runs a Cox model on gender and expression with survival!

coxall <- coxph(Surv(time, event) ~ gend*exps, data = cancer)

summary(coxall)

#gender based visualisation

fit <- survfit(Surv(time, event) ~ gend, data = cancer)

# Visualize with survminer

ggsurvplot(fit, data = cancer, risk.table = TRUE, pval = TRUE, conf.int = TRUE, xlim = c(0,300), break.time.by = 30, # break X axis in time intervals by 500.

ggtheme = theme_minimal(), risk.table.y.text.col = T, risk.table.y.text = FALSE, legend.title = "Sex",

legend.labs = c("Male", "Female"),)

#Expression data hazard ratio - interpret carefully!

notibsall <- as.data.frame(cancer)

res.cox <- coxph(Surv(time, event) ~ exps, data = notibsall, model=TRUE, x=TRUE)

summary(res.cox)

hrall <- smoothHR(data=notibsall, coxfit=res.cox)

plot(hrall, predictor="exps", prob=0, conf.level=0.95, main = "Log Hazard of YBX1 expression", xlab="Expression level")
