# Supplementary material for: In silico analysis of overall survival with YBX1 in male and female solid tumours
Source: Sci Rep. 2024 Mar 27;14:7218. doi: 10.1038/s41598-024-57771-y (PMC10973514; doi:10.1038/s41598-024-57771-y)
Supplement: Supplementary file 2 — Supplementary Table 1. [file 41598_2024_57771_MOESM2_ESM.docx]

Supplementary Table 1: List of X-linked genes co-expressing with YB-1 in both male and female patients.

| Gene | Cytoband | Correlation Coefficient Male | q-value  Male | Correlation Coefficient Female | q-value Female |
| --- | --- | --- | --- | --- | --- |
| AMOT | Xq23 | -0.30 | 0.0000027 | -0.35 | 0.004 |
| ARMCX3 | Xq22.1 | -0.27 | 0.0000420 | -0.29 | 0.021 |
| ARSD | Xp22.33 | -0.34 | 0.0000001 | -0.41 | 0.001 |
| ATP7A | Xq21.1 | -0.28 | 0.0000205 | -0.40 | 0.001 |
| BCLAF3 | Xp22.12 | -0.29 | 0.0000060 | -0.37 | 0.002 |
| CCDC160 | Xq26.2 | -0.36 | 0.0000000 | -0.35 | 0.004 |
| DKC1 | Xq28 | 0.36 | 0.0000000 | 0.54 | 0.000 |
| EIF2S3 | Xp22.11 | 0.30 | 0.0000022 | 0.30 | 0.016 |
| EMD | Xq28 | 0.31 | 0.0000016 | 0.33 | 0.006 |
| FTSJ1 | Xp11.23 | 0.32 | 0.0000004 | 0.35 | 0.004 |
| GLOD5 | Xp11.23 | -0.38 | 0.0000000 | -0.39 | 0.001 |
| HPRT1 | Xq26.2-q26.3 | 0.26 | 0.0000701 | 0.30 | 0.016 |
| INE1 | Xp11.3 | -0.28 | 0.0000132 | -0.27 | 0.034 |
| IQSEC2 | Xp11.22 | -0.26 | 0.0000507 | -0.42 | 0.000 |
| IRAK1 | Xq28 | 0.26 | 0.0000833 | 0.45 | 0.000 |
| KIF4A | Xq13.1 | 0.28 | 0.0000106 | 0.39 | 0.001 |
| KLF8 | Xp11.21 | -0.28 | 0.0000148 | -0.33 | 0.008 |
| MAGIX | Xp11.23 | -0.31 | 0.0000008 | -0.26 | 0.039 |
| MID1 | Xp22 | -0.26 | 0.0000901 | -0.36 | 0.003 |
| NAA10 | Xq28 | 0.30 | 0.0000026 | 0.43 | 0.000 |
| OPHN1 | Xq12 | -0.32 | 0.0000006 | -0.36 | 0.003 |
| PGK1 | Xq21.1 | 0.27 | 0.0000391 | 0.44 | 0.000 |
| POF1B | Xq21.1 | -0.28 | 0.0000162 | -0.36 | 0.003 |
| PRDX4 | Xp22.11 | 0.25 | 0.0001251 | 0.41 | 0.001 |
| PRPS1 | Xq22.3 | 0.29 | 0.0000063 | 0.36 | 0.003 |
| RPS6KA6 | Xq21.1 | -0.31 | 0.0000014 | -0.43 | 0.000 |
| SHROOM4 | Xp11.22 | -0.28 | 0.0000153 | -0.37 | 0.002 |
| SLC25A5 | Xq24 | 0.26 | 0.0000657 | 0.51 | 0.000 |
| STS | Xp22.31 | -0.28 | 0.0000122 | -0.37 | 0.002 |
| SYTL4 | Xq22.1 | -0.26 | 0.0000618 | -0.32 | 0.009 |
| SYTL5 | Xp11.4 | -0.25 | 0.0001228 | -0.34 | 0.005 |
| TIMM8A | Xq22.1 | 0.27 | 0.0000378 | 0.52 | 0.000 |
| TSPAN6 | Xq22.1 | -0.25 | 0.0001055 | -0.37 | 0.002 |
| UTP14A | Xq26.1 | 0.36 | 0.0000000 | 0.58 | 0.000 |
| VGLL1 | Xq26.3 | -0.42 | 0.0000000 | -0.41 | 0.000 |
| ZC3H12B | Xq11.2-q12 | -0.26 | 0.0000560 | -0.36 | 0.003 |
| ZNF41 | Xp11.3 | -0.27 | 0.0000340 | -0.30 | 0.014 |
