# Supplementary material for: In silico analysis of overall survival with YBX1 in male and female solid tumours
Source: Sci Rep. 2024 Mar 27;14:7218. doi: 10.1038/s41598-024-57771-y (PMC10973514; doi:10.1038/s41598-024-57771-y)
Supplement: Supplementary file 3 — Supplementary Table 2. [file 41598_2024_57771_MOESM3_ESM.docx]

Supplemental table 2. X-linked genes significantly correlated with YB-1 in female bladder cancer patients.

| Genes | Cytoband | Correlation | p value | q value |
| --- | --- | --- | --- | --- |
| VBP1 | Xq28 | 0.400 | 0.000 | 0.001 |
| VMA21 | Xq28 | 0.345 | 0.000 | 0.005 |
| IDH3G | Xq28 | 0.310 | 0.001 | 0.012 |
| UBL4A | Xq28 | 0.302 | 0.002 | 0.015 |
| RPL10 | Xq28 | 0.301 | 0.002 | 0.016 |
| CCNQ | Xq28 | 0.294 | 0.002 | 0.019 |
| HAUS7 | Xq28 | 0.289 | 0.003 | 0.021 |
| G6PD | Xq28 | 0.268 | 0.005 | 0.034 |
| MPP1 | Xq28 | 0.266 | 0.006 | 0.036 |
| SNORA56 | Xq28 | 0.265 | 0.006 | 0.037 |
| FATE1 | Xq28 | 0.256 | 0.008 | 0.045 |
| RTL8B | Xq26.3 | -0.286 | 0.003 | 0.023 |
| RBMX | Xq26.3 | 0.259 | 0.007 | 0.042 |
| ZNF449 | Xq26.3 | -0.258 | 0.007 | 0.043 |
| AIFM1 | Xq26.1 | 0.329 | 0.001 | 0.008 |
| APLN | Xq26.1 | 0.290 | 0.003 | 0.021 |
| MCTS1 | Xq24 | 0.402 | 0.000 | 0.001 |
| NKAP | Xq24 | 0.270 | 0.005 | 0.033 |
| TRPC5 | Xq23 | -0.273 | 0.005 | 0.031 |
| NUP62CL | Xq22.3 | 0.282 | 0.003 | 0.025 |
| NRK | Xq22.3 | -0.274 | 0.004 | 0.030 |
| IRS4 | Xq22.3 | -0.253 | 0.009 | 0.048 |
| GPRASP1 | Xq22.1 | -0.391 | 0.000 | 0.001 |
| GLA | Xq22.1 | 0.327 | 0.001 | 0.008 |
| CSTF2 | Xq22.1 | 0.301 | 0.002 | 0.016 |
| CENPI | Xq22.1 | 0.283 | 0.003 | 0.024 |
| NOX1 | Xq22.1 | -0.255 | 0.008 | 0.046 |
| DACH2 | Xq21.2 | -0.257 | 0.008 | 0.044 |
| PGAM4 | Xq21.1 | 0.324 | 0.001 | 0.008 |
| ZNF711 | Xq21.1 | -0.292 | 0.002 | 0.020 |
| COX7B | Xq21.1 | 0.264 | 0.006 | 0.038 |
| ABCB7 | Xq13.3 | 0.325 | 0.001 | 0.008 |
| PBDC1 | Xq13.3 | 0.273 | 0.005 | 0.031 |
| MAGEE2 | Xq13.3 | -0.251 | 0.009 | 0.050 |
| FOXO4 | Xq13.1 | -0.407 | 0.000 | 0.001 |
| PHKA1 | Xq13.1 | -0.383 | 0.000 | 0.001 |
| ERCC6L | Xq13.1 | 0.318 | 0.001 | 0.010 |
| MED12 | Xq13.1 | -0.316 | 0.001 | 0.010 |
| EFNB1 | Xq13.1 | 0.312 | 0.001 | 0.012 |
| PJA1 | Xq13.1 | 0.310 | 0.001 | 0.012 |
| FAM226B | Xq13.1 | -0.306 | 0.001 | 0.014 |
| NONO | Xq13.1 | 0.278 | 0.004 | 0.027 |
| NHSL2 | Xq13.1 | -0.259 | 0.007 | 0.042 |
| LAS1L | Xq12 | 0.259 | 0.007 | 0.043 |
| NLGN4X | Xp22.32-p22.31 | -0.283 | 0.003 | 0.025 |
| SLC25A6 | Xp22.32 and Yp11.3 | 0.362 | 0.000 | 0.003 |
| NHS | Xp22.2-p22.13 | -0.354 | 0.000 | 0.004 |
| HCCS | Xp22.2 | 0.418 | 0.000 | 0.000 |
| REPS2 | Xp22.2 | -0.302 | 0.002 | 0.015 |
| OFD1 | Xp22.2 | -0.275 | 0.004 | 0.029 |
| ARHGAP6 | Xp22.2 | -0.261 | 0.007 | 0.040 |
| FANCB | Xp22.2 | 0.253 | 0.009 | 0.048 |
| RS1 | Xp22.13 | -0.279 | 0.004 | 0.027 |
| PDHA1 | Xp22.12 | 0.417 | 0.000 | 0.000 |
| EIF1AX | Xp22.12 | 0.277 | 0.004 | 0.028 |
| PTCHD1 | Xp22.11 | -0.397 | 0.000 | 0.001 |
| APOO | Xp22.11 | 0.368 | 0.000 | 0.002 |
| SMS | Xp22.11 | 0.356 | 0.000 | 0.003 |
| ACOT9 | Xp22.11 | 0.317 | 0.001 | 0.010 |
| TAB3 | Xp21.2 | -0.288 | 0.003 | 0.022 |
| CYBB | Xp21.1-p11.4 | 0.252 | 0.009 | 0.049 |
| XK | Xp21.1 | -0.299 | 0.002 | 0.016 |
| ZNF81 | Xp11.23 | -0.369 | 0.000 | 0.002 |
| PLP2 | Xp11.23 | 0.361 | 0.000 | 0.003 |
| ZNF630 | Xp11.23 | -0.355 | 0.000 | 0.004 |
| SUV39H1 | Xp11.23 | 0.348 | 0.000 | 0.004 |
| CCDC120 | Xp11.23 | -0.343 | 0.000 | 0.005 |
| USP27X-DT | Xp11.23 | -0.305 | 0.001 | 0.014 |
| ZNF182 | Xp11.23 | -0.302 | 0.002 | 0.015 |
| SLC38A5 | Xp11.23 | 0.286 | 0.003 | 0.023 |
| USP27X | Xp11.23 | -0.255 | 0.008 | 0.046 |
| PQBP1 | Xp11.23 | 0.253 | 0.009 | 0.048 |
| HSD17B10 | Xp11.22 | 0.310 | 0.001 | 0.012 |
| TSPYL2 | Xp11.22 | -0.293 | 0.002 | 0.019 |
| PHF8 | Xp11.22 | -0.286 | 0.003 | 0.023 |
| FAAH2 | Xp11.21 | -0.315 | 0.001 | 0.011 |
| SPIN3 | Xp11.21 | -0.261 | 0.007 | 0.040 |
| PAGE5 | Xp11.21 | -0.258 | 0.007 | 0.044 |
