# Supplementary material for: In silico analysis of overall survival with YBX1 in male and female solid tumours
Source: Sci Rep. 2024 Mar 27;14:7218. doi: 10.1038/s41598-024-57771-y (PMC10973514; doi:10.1038/s41598-024-57771-y)
Supplement: Supplementary file 4 — Supplementary Table 3. [file 41598_2024_57771_MOESM4_ESM.docx]

Supplemental table 3. X-linked genes significantly correlated with YB-1 in male bladder cancer patients.

| \| **Genes** \| **Cytoband** \| **Correlation** \| **p value** \| **q value** \| \| --- \| --- \| --- \| --- \| --- \| \| IL9R \| Xq28 and Yq12 \| -0.27 \| <0.001 \| <0.001 \| \| LINC00893 \| Xq28 \| -0.26 \| <0.001 \| <0.001 \| \| LINC00894 \| Xq28 \| -0.25 \| <0.001 \| <0.001 \| \| MAP7D3 \| Xq26.3 \| 0.34 \| <0.001 \| <0.001 \| \| ZNF75D \| Xq26.3 \| -0.33 \| <0.001 \| <0.001 \| \| MOSPD1 \| Xq26.3 \| -0.29 \| <0.001 \| <0.001 \| \| XIAP \| Xq25 \| -0.32 \| <0.001 \| <0.001 \| \| RNF128 \| Xq22.3 \| -0.30 \| <0.001 \| <0.001 \| \| GJB1 \| Xq13.1 \| -0.31 \| <0.001 \| <0.001 \| \| MSN \| Xq12 \| 0.28 \| <0.001 \| <0.001 \| |
| --- | --- | --- | --- | --- | --- | --- | --- | --- | --- | --- | --- | --- | --- | --- | --- | --- | --- | --- | --- | --- | --- | --- | --- | --- | --- | --- | --- | --- | --- | --- | --- | --- | --- | --- | --- | --- | --- | --- | --- | --- | --- | --- | --- | --- | --- | --- | --- | --- | --- | --- | --- | --- | --- | --- | --- |
